# Chitosan-tripolyphosphate-tannic acid cryogels as a biocompatible adsorbent for the removal of Cu^2+^ ions

**DOI:** 10.1098/rsos.242274

**Published:** 2025-08-06

**Authors:** Jaya Hardi, Tristan Tijsseling, Hayato Takase, Masahiro Yoshida, Koichiro Shiomori, Hideki Matsune

**Affiliations:** ^1^ University of Miyazaki, Miyazaki, Japan; ^2^ Department of Chemistry, Tadulako University, Palu, Indonesia; ^3^ Avans University of Applied Sciences in Breda, Breda, The Netherlands; ^4^ Department of Chemical Engineering, Kagoshima University, Kagoshima, Japan; ^5^ Department of Applied Chemistry, University of Miyazaki, Miyazaki, Miyazaki Prefecture, Japan

**Keywords:** cryogel, chitosan, tannic acid, tripolyphosphate, adsorbent, Cu^2+ ^ions

## Abstract

A chitosan (CH) cryogel is a supermacropore material that can be used as an adsorbent. However, its utilization as a metal adsorbent is limited due to its low stability in acidic conditions. A CH cryogel has been modified using tripolyphosphate (TPP) and tannic acid (TA) as biocompatible cross-linkers. TPP increases the stability of the cryogels in acidic solutions, and TA increases their mechanical properties. CH-TPP, CH-TPP2.5-TA, CH-TPP5-TA and CH-TPP7.5-TA cryogels were applied for the adsorption of Cu^2+^ ions. The CH-TPP-TA cryogels reached optimum conditions at pH 5.0 with a removal efficiency of 91–96%. The adsorption of Cu^2+^ ions by CH-TPP-TA cryogels fits the nonlinear Freundlich isotherm, or Cu^2+^ is adsorbed on the heterogeneous cryogels’ surfaces. CH-TPP5-TA has an adsorption capacity up to 109 mg g^−1^ at an initial Cu^2+^ concentration of 500 mg l^−1^. The adsorption kinetics of Cu^2+^ ions follows pseudo-second order. It is interpreted that the adsorption mechanism is generally through chemical interactions. Copper elements are proven to be adsorbed by the cryogel, which is detected by energy-dispersive X-ray analysis. The photographic results show that Cu^2+^ ions can be adsorbed into the inner layer of the cryogel. Finally, the CH-TPP-TA cryogel is promising to be used as a biocompatible adsorbent for copper.

## 1. Introduction

Environmental pollution is one of the significant problems of our time. Industrial activities are increasingly expanding and causing large amounts of wastewater to be discharged into the environmental water [1]. Copper (Cu) is one of the most common pollutants in industrial waste, especially in the mining and electronics industries [2,3]. Cu is a heavy metal with the third highest level of toxicity after mercury (Hg) and cadmium (Cd) [4,5]. Its poisoning will cause intestinal and stomach disorders, kidney failure, inhibit the activity of microbes in metabolism and Wilson’s disease [5]. Therefore, it is vital to control and remove copper from water.

Various methods have been developed to overcome Cu metal pollution in water by methods including precipitation, ion exchange, filtration, electrochemistry and adsorption techniques. Each technique used has its advantages and disadvantages. In this research, we used adsorption because it is a low-cost, highly efficient and simple design with wide applications [1,2,6].

Many adsorbents have been developed from inorganic and organic compounds, such as zeolites [7], activated carbon [8] and carbohydrate group compounds [9]. Carbohydrates are the most abundant type of biopolymer in nature. Therefore, they are easy to use as biocompatible adsorbents. Carbohydrate derivatives are plentiful and used as adsorbents, such as cellulose [10], starch [11], pectin [12] and chitosan [13]. Chitosan (CH) was chosen in this study because it has an easily modified, inexpensive, easy-to-extract and non-toxic structure [14,15]. CH-based adsorbents have been widely applied for metal adsorption, especially Cu(II) metal from aqueous solutions, and can remove more than 90% of Cu^2+^ ions [16,17]. CH is a natural heteropolysaccharide produced by the deacetylation of chitin [18]. CH has high percentages of amine and hydroxyl groups and performs well in complexing or chelating metal ions. The degree of deacetylation (DD) describes the number of N-acetyl groups converted into amines. A high DD value, interpreted as having a value greater than 70%, will increase the performance of CH in adsorbing organic and inorganic compounds [18,19]. The amine group of CH plays an essential role in binding metal ions. With a DD value of 86.9%, CH was reported to adsorb up to 84.09% of Cu(II) [20]. In another study, CH combined with hydroxyapatite could adsorb up to 71.5 mg g^−1^ of copper ions at pH 5.5 [21]. Magnetic CH microspheres (DD 86.1%) were reported to adsorb 62.3 mg g^−1^ of Cu(II) from wastewater [22]. The amine group of CH is easily protonated in an acidic solution; therefore, its application is limited [23,24].

Chemical compounds such as cross-linking agents can be added to the CH structure to increase its stability in an acidic environment. CH was previously applied to metal adsorption, for which it was developed using glutaraldehyde as a cross-linking agent [25]. Good elasticity properties of the cross-linked gel are expected to reduce the swelling effect on the gel and avoid permanent deformation of the gel shape. This makes the gel durable when applied to continuous adsorption. However, glutaraldehyde is a type of cross-linker that is not biocompatible, so it is unsuitable for applications including drinking water treatment. Therefore, a cross-linking agent should be safe. Sodium tripolyphosphate (Na_5_P_3_O_10_) has been reported as one of the safe ingredients for humans and even listed as a generally recognized as safe (GRAS) food additive by the Food and Drug Administration (FDA) [26]. In our study, we have chosen tripolyphosphate (TPP) as a cross-linking agent because the composite of CH-TPP has been reported to have superior biocompatible and mechanical properties compared with CH-glutaraldehyde [27]. TPP has phosphate groups that can interact ionically with CH and form cross-links [28]. The abundant ionic interactions in the CH structure make it more resistant to an acidic environment [14]. The interaction between CH-NH_3_^+^ and TPP-O^−^ groups is known as a Coulombic interaction. The ionic interaction of CH-TPP is stable in acidic solutions, and TPP will begin to be released at an alkaline pH level [29]. Accordingly, the CH-TPP complex is ideal for metal ions’ adsorption process, which generally has optimum adsorption conditions at pH 5−7. In addition, tannic acid (TA) is used in this study to improve the mechanical properties of CH-TPP composites. CH and TA have previously been reported to react well to form multifunctional composite films [15]. TA has a polyphenol component capable of forming hydrogen bonds and interacting ionically and hydrophobically with CH (electronic supplementary material, figure S1). TA has been identified as strengthening the CH cryogel matrix by forming hydrogen bonds [30]. Other studies have also reported that hydrogen bonds in acrylate–acrylamide hydrogels increase the mechanical strength of the gel and inhibit gel swelling [31]. The high mechanical strength of the gel is helpful in a continuous adsorption system. Another advantage of TPP and TA as cross-linking agents is that they are non-toxic and are therefore known as ‘biocompatible cross-linkers’ [27,30,32].

The CH-TPP-TA cryogel is the newest type of material based on natural compounds. In our previous research, we created a form of cryogel with interactions between CH, TPP and TA. The cryogel is a spongy porous material with high biocompatibility and a flexible structure. The CH-TPP-TA cryogel has a ‘supermacropore’ structure [30]. Supermacropores have an average pore diameter of 50 μm [33]. Cryogels are made via the cryogelation technique controlled by freezing and thawing [34]. It has superior properties, including mechanically robust, elastic networks, easy storage, good flow-through qualities and easy handling compared with those of hydrogels that are usually formed at room temperature [35–38]. The macroporous structure of the cryogel facilitates water flow through a short diffusion route, leading to the transfer and adsorption of substances occurring more quickly than in the hydrogel form [33,36]. Thereby, it is widely applied as a metal adsorbent [39]. Although CH cryogel, as an adsorbent, has been generally developed using glutaraldehyde cross-linkers [25,40], we apply a cryogel of CH-TPP-TA for metal ion adsorption. Our previous research showed that cryogel from a combination of CH 1%, TPP (2.5, 5.0 and 7.5%) and TA 5% had higher porosity and were more resistant to gel swelling than CH-TPP cryogel (cryogel without TA) [30]. This research is an initial study using CH-TPP-TA as a biocompatible adsorbent to remove metal ions. Cu^2+^ ion is used as a model of metal ions to study the adsorption ability of the cryogels. Cu^2+^ ion is classified into the intermediate group according to the hard and soft acids and bases (HSAB) theory. Thus, it can interact with strong and weak base ligands [41,42]. CH-TPP-TA has amine groups as an intermediate base ligand and hydroxyl groups as a strong base ligand. The amino and hydroxyl functional groups from CH and the hydroxyl groups from TA are thought to increase the effectiveness of Cu^2+^ ion adsorption. Using TPP and TA as cross-linker agents for Cu^2+^ ion adsorption is a novelty in our research. The CH-TPP-TA cryogel is a promising biocompatible adsorbent.

## 2. Material and methods

### 2.1. Materials

Cryogel was synthesized using CH (1st grade, DD ≥ 80%), sodium tripolyphosphate, acetic acid (1st grade, 99%) obtained from Fujifilm Wako and tannic acid (1st grade) from Sigma-Aldrich. Copper(II) chloride dihydrate (1st grade, 97%) from Fujifilm Wako was used as an adsorbate solution. Other chemical materials were copper standard solution (Fujifilm Wako, 990–1010 mg l^−1^), sodium hydroxide (Fujifilm Wako 1st grade, greater than or equal to 93%), hydrochloric acid (Sigma-Aldrich 1st grade, 10%), ethanol (99%) and deionized water (DI-water).

### 2.2. Synthesis of the chitosan cryogels

Cryogels consisting of TPP (2.5, 5 and 7.5 wt%) and TA (1 wt%) were prepared according to our previous method [30]. Typically, CH 1 wt% in 25 ml acetic acid 2 v/v% and sodium tripolyphosphate solution of 1.31 ml were homogenized at 13 500 r.p.m for 10 min, then stirred for 1 h in an ice bath. The dispersion was placed in a plastic syringe and frozen at −15°C for 48 h. The cryogels were thawed and washed with 0.1 M NaOH aq., DI water and ethanol. The CH-TPP cryogels were immersed in 15 ml of TA solution at room temperature for 4 h [25,30,43,44]. The CH-TPP-TA cryogels were washed with DI water and ethanol and freeze-dried (TAITEC VD−250F) for 24 h [30,45]. The calculated percentages of the three main components in the final composite are shown in electronic supplementary material, table S1.

### 2.3. Batch adsorption procedure

Adsorption of Cu^2+^ ions by 15 mg cryogels was carried out in a batch reactor containing 10 ml of copper chloride solution in a water bath shaker (Shimadzu SB-220) at a temperature of 303 K with constant stirring at 50 r.p.m. [46,47]. Cryogels were added to Cu^2+^ ion solutions with differences in pH, initial concentration of Cu^2+^ ions and adsorption time. The study of the effect of pH on Cu^2+^ ion adsorption by cryogels at pH 1−6 was adjusted with hydrochloric acid and sodium hydroxide. Adsorption isotherms were determined at different initial Cu^2+^ ion concentrations (50–500 mg l^−1^). Adsorption kinetics were obtained based on the study of adsorption time (10−1440 min) using 30 ml of metal solution and 60 mg of cryogels. Inductively coupled plasma spectrometer (ICPS-8100, Shimadzu) analysed the concentration of Cu^2+^ ions in the filtrate at a wavelength of 327.4 nm; then it was used to calculate adsorption capacity (*q*_e_) and removal efficiency (electronic supplementary material, table S2).

The adsorption isotherm is determined using the Langmuir, Freundlich, nonlinear Langmuir, nonlinear Freundlich and Temkin isotherm models (electronic supplementary material, table S2). Adsorption isotherms were selected based on the highest coefficient of determination (*R^2^*) [1,48]. The Temkin isotherm model describes the interaction between adsorbates, indirectly affecting adsorption. The linear Langmuir, Freundlich and Temkin isotherms use equations (2.1)–(2.3), respectively,


(2.1) $$\frac{C_{e}}{q_{e}}=\frac{C_{e}}{q_{m}}+\frac{1}{k_{L}},$$

(2.2) $$ln(q_{e})=(1/n)lnC_{e}+ln(k_{F})$$

and


(2.3) $$q_{e}=BlnC_{e}+BlnA_{T},$$

where *q*_e_ (mg g^−1^) is the adsorption capacity of Cu^2+^ ions, *q*_m_ (mg g^−1^) is the maximum adsorption capacity of Cu^2+^ ions, *C*_e_ (mg l^–1^) is the equilibrium concentration of Cu^2+^ in the solution, *k*_L_ is the Langmuir constant, *k*_F_ is the Freundlich constant and 1/*n* is a predictor of favourable adsorption. *B* and *A*_T_ are Temkin parameters [49,50].

Adsorption kinetics were determined using pseudo-first and pseudo-second order ((PFO, PSO); electronic supplementary material, table S2). Adsorption kinetics were selected based on the highest coefficient of determination (*R^2^*) from the linearized PFO and PSO curves, as shown in equations (2.4) and (2.5), respectively [1,48],

(2.4) $$log(q_{e}-q_{t})=log(q_{e})-(\frac{k_{1}}{2.303})\times t$$

and

(2.5) $$\frac{t}{q_{t}}=\frac{t}{q_{e}}+\frac{1}{k_{2}\times q_{e}^{2}},$$

where *q_t_* (mg g^−1^) is the time adsorption capacity, *k*_1_ (min^−1^) is the first-order rate coefficient, *k*_2_ (min^−1^) is the second-order rate coefficient and *t* (min) is the adsorption time.

### 2.4. Characterization

The mechanical properties were determined at room temperature on swollen cryogels with a 9−12 mm diameter and approximately 9−10 mm length using an Orientec Universal Testing Machine (RTC-1210A). To ensure complete contact between the cryogel surfaces and the compression plates of the testing machine, an initial force of 0.5 N was applied before conducting each analysis. The Young’s modulus was obtained from the slope of the linear compressive stress versus strain curve, and the yield strength was determined using the 0.2% offset yield strength method [51].

The surface morphology of the CH cryogel before and after Cu^2+^ adsorption was observed using scanning electron microscopy (SEM, Hitachi TM1000). The distribution of Cu^2+^ ions on the surface of the cryogel was mapped using SEM–energy-dispersive X-ray (SEM-EDX, Hitachi SU3500-EDAX Genesis-APEX2), which had previously been coated with gold (Au) metal (ion sputtering devices, Hitachi E-1030 and E-1045). The adsorption of Cu^2+^ ions from the outer layer to the inner layer of the cryogel was photographed using a stereomicroscope system (Olympus SZX7 and WRAYCAN EL310). Cryogels’ images were compared before and after the adsorption of Cu^2+^ ions.

The UV–visible (UV-Vis) diffuse reflection spectra of cryogel–Cu complex were collected using a UV-Vis spectrophotometer (JASCO V-750) equipped with a 60 mm integrating sphere (ISV-922) for the investigation of the complexation mechanism of Cu^2+^ with TA and CH in the gel form. Meanwhile, the solution state was investigated using a transmission mode (photometric range of −4–4 Abs).

### 2.5. Statistical analysis

All adsorption capacities and standard deviations from three experiments in the study were estimated using Microsoft Excel 2016. Standard deviations and error bars are included in all adsorption data. Calculations of adsorption isotherms and kinetics were performed using the OriginPro 2024b (Learning Edition) program.

## 3. Results and discussion

### 3.1. Synthesis of chitosan cryogels

**Figure 1.**
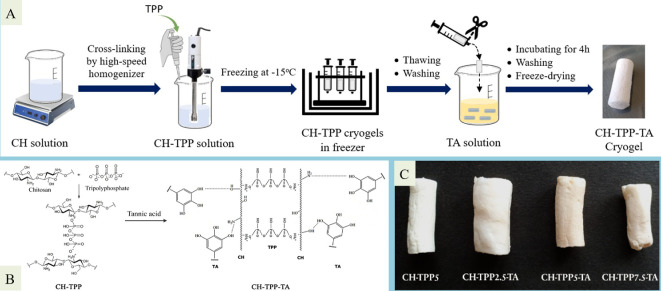
Schematic illustration of cryogel synthesis: (A) chemical reaction in the cryogel synthesis (B) and appearance of cryogels (C).

Cryogel was synthesized by interacting with CH, TPP and TA (figure 1A). TPP, as a cross-linker, will interact ionically with the CH chain. At the same time, TA can act as a cross-linker and coating agent that interacts with CH through the formation of hydrogen bonds, ionic interactions and London forces (figure 1B). The effectiveness of the CH-TPP-TA cryogel formation method through the immersion process in a TA solution was determined using a UV-Vis spectrophotometer at a maximum TA wavelength of 235 nm. TA in cryogel has a yield of more than 50% (electronic supplementary material, figure S2). This amount is enough to strengthen the CH cryogel. Therefore, it does not quickly swell in aqueous solutions.

The four types of cryogels produced have different appearances. CH-TPP cryogel turned from colourless to pale brown with the complexation of TA (figure 1C). The brown colour comes from TA compounds. In our previous study, we characterized the infrared spectrum of the CH-TPP-TA cryogel. The stretching vibrations of C=O, C=C aromatic, C-O ester and C-O-C ether at wavenumbers 1712, 1615, 1445, 1204 and 1164 cm^−1^ are the characteristic spectra of TA. Two absorption peaks of PO_2_ and P-O-P at wavenumbers of 1158 and 896 cm^−1^ have proven the cross-linking of TPP and CH [30]. This spectrum demonstrates that CH-TPP-TA composites have been formed. At low TPP concentrations, the cryogel volume is more significant due to the formation of macropores in the cryogel, while at high concentrations, the cryogel volume is smaller. At low TPP concentrations, the degree of cross-linking is lower. Therefore, ice nucleation quickly occurs during the freeze-drying process. Each cryogel pore results from the growth of several ice grains [52]. Due to the lack of added cross-linking agents, long CH polymer chains will also quickly form intramolecular hydrogen bonds during freeze-drying. Thereby, the crystal growth rate occurs quickly [53]. Sublimation of ice crystals will form macropores in the drying phase. Essential information we have found in previous studies is that high TPP concentration reduces the pore size of the cryogel [30]. We suspect that as the degree of cross-linking increases, the growth of ice crystals is inhibited, ultimately resulting in a smaller pore structure. TPP with a high concentration will also hinder the interaction of CH and TA. Therefore, the brown colour of the cryogel will fade as the TPP concentration increases. Although a high amount of TPP has a negative effect on the cryogel, the absence of TPP makes it extremely difficult for the cryogel structure to form. We attempted to create it from CH-TA, but the cryogel structure failed to form after freezing.

**Figure 2.**
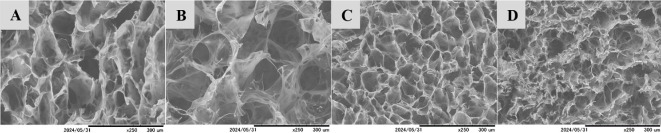
Morphology of CH-TPP5 (A), CH-TPP2.5-TA (B), CH-TPP5-TA (C), and CH-TPP7.5-TA (D) cryogels.

All the cryogels formed pores (figure 2). Our previous study found that CH-TPP2.5-TA and CH-TPP5-TA cryogels have an average pore diameter of approximately 50 μm (‘supermacropores’). CH-TPP2.5-TA, CH-TPP5-TA and CH-TPP7.5-TA have diameter pores of 47.6, 56.8 and 27.2 μm, respectively. The porosity values of these cryogels were more than 90% [30]. Dry cryogel with a high TPP concentration (percentage of TPP in CH-TPP7.5-TA more than 23%) produces brittle materials. This means a high TPP amount in CH cryogel will increase the degree of cross-linking and eventually form a compact structure. Compact materials will lead to decreased elasticity and will have difficulty deforming [54]. Furthermore, the high interaction between TPP and CH will reduce the interaction between CH and TA. We assumed that TA has an essential role in increasing the matrix strength and the deformation of the cryogel [30]. TA has been proven to maintain the cryogel’s shape from the swelling process in aqueous solutions.

**Figure 3.**
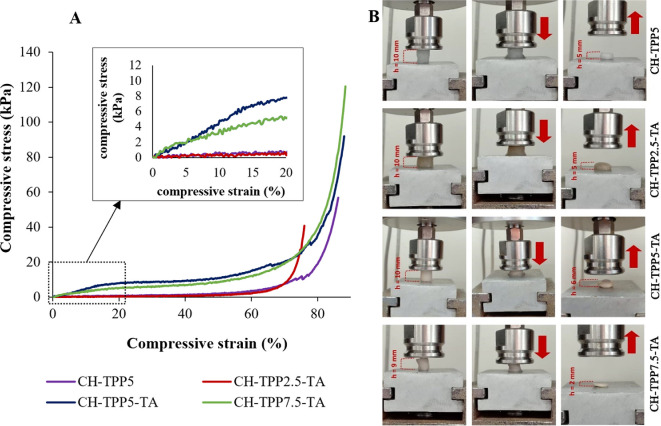
The compressive stress−strain profile (A) and compressibility images (B) of swollen cryogels.

The swelling ratio values of the four cryogels (CH-TPP5, CH-TPP2.5-TA, CH-TPP5-TA and CH-TPP7.5-TA) were less than 12 g g^−1^ gel (table 1), which is lower than the swelling ratio of cellulose polysaccharide-based hydrogels of 16−18 g g^−1^ [55]. CH cryogels with linkers are resistant to swelling. We were interested in explaining the effect of the cross-linking agent on the mechanical properties of the cryogel. Quite clear differences in Young’s modulus were produced from the compressive test among the four cryogels (figure 3A and electronic supplementary material, figure S3). The Young’s modulus values of CH-TPP5-TA and CH-TPP7.5-TA were higher than those of CH-TPP5 and CH-TPP2.5-TA (table 1). In the swollen state, CH-TPP5-TA had the highest elastic modulus of 0.478 kPa, followed by CH-TPP7.5-TA of 0.279 kPa. The Young’s modulus value is inversely proportional to the swelling ratio value [56]. Interestingly, CH-TPP7.5-TA did not recover its shape well after compression (figure 3B). We assume that when the amount of TPP is excessive, not all of its phosphate groups are cross-linked with CH. In a swelling state, in addition to the dissociation of intermolecular hydrogen bonds of CH [55], the free phosphate groups of TPP also form hydrogen bonds with water molecules. During compression, water molecules are released from the cryogel, and the CH matrix becomes dense. At the same time, free phosphate groups of TPP will form new cross-links with CH, which results in a denser cryogel structure that is not recoverable to its original shape. More apparent results were obtained in dry conditions of cryogels. CH-TPP7.5-TA cryogel is more brittle than other cryogels. The combination of CH-TPP5-TA (component ratio of 63 : 16 : 21) was ideal, with a dry cryogel structure that was not brittle and had a low swelling ratio. The Young’s modulus number produced in this study is lower than other CH cryogels using α-ketoglutaric acid silicate binders of 0.4−1.7 kPa [57]. It illustrates that the cryogel in this study was more elastic, or not stiff. The yield strength of CH-TPP5-TA has the highest value of 6.78 kPa, which means that the cryogel structure can withstand deformation without breaking or failing. Although CH cryogel without TA can experience shape recovery, its easy-to-swell nature can facilitate gel damage during continued use. This indicates that TA can increase the strength of the CH gel matrix. Therefore, CH-TPP5-TA has the potential to be used in continuous adsorption systems because it has flexibility and non-brittleness.

**Table 1.** The mechanical properties of CH cryogels.

cryogels	swelling ratio (g g^−1^) [30]	young’s modulus (kPa)	yield strength (kPa)
CH-TPP5	11.1 ± 2.58	0.043	0.957
CH-TPP2.5-TA	9.33 ± 0.10	0.030	0.598
CH-TPP5-TA	7.22 ± 0.08	0.478	6.784
CH-TPP7.5-TA	7.16 ± 0.26	0.279	5.483

### 3.2. Effect of pH solution

**Figure 4.**
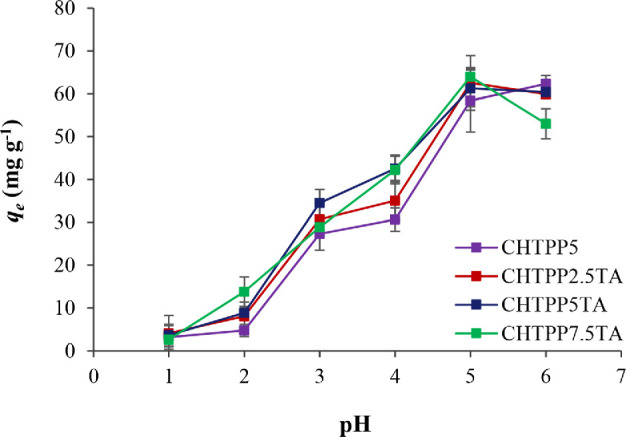
The effect of pH on the adsorption capacity.

The application of CH cryogel was carried out on the adsorption of Cu^2+^. A *q*_e_ value increases with the increase in pH (figure 4). The optimum pH of cryogels on Cu^2+^ adsorption in aqueous solutions was generally pH 5, with a *q*_e_ value of 61−63 mg g^−1^ of cryogels (electronic supplementary material, table S3). All the cryogels have a deficient adsorption capacity in a strongly acidic solution (pH 1), which was caused by the protonation of amine groups of CH. CH-TPP has the lowest *q*_e_ at pH 1 (removal efficiency less than 5%) and pH 2 (removal efficiency less than 8%). In addition, the cryogels dissolved in the acid solution after incubation for 24 hours at pH 1 or lower [14,58]. CH cryogel without TA was dissolved in an acidic solution, even at pH 2, but it retained its structure at pH 3−6. However, CH-TPP-TA was stable in solutions at pH 2−6. This demonstrates that TA has a dual role as a cross-linker and coating agent, increasing CH’s resistance to acid. The number of free amine groups of CH increases with increasing pH of the solution. They bind Cu^2+^ ions more easily than in the protonated state. Free amine groups of CH effectively complex Cu^2+^ ions up to pH 5. However, at pH 6 or more, Cu^2+^ ions will begin to precipitate as Cu(OH)_2_ [59]. The most important thing to mention is that industrial wastewater generally has a pH of approximately 5 [60]. The pH 5 area also prevents the release of TPP from the CH-TPP composite. Protonated CH will precipitate at alkaline pH due to the deprotonation or neutralization process. Meanwhile, the ionization of TPP depends on pH because it has a different pKa value of 0.9−7.7 [61,62]. Furthermore, the alkaline pH of the solution causes the release of TPP from the CH-TPP composite as a result of the neutralization of CH [29,61].

**Figure 5.**
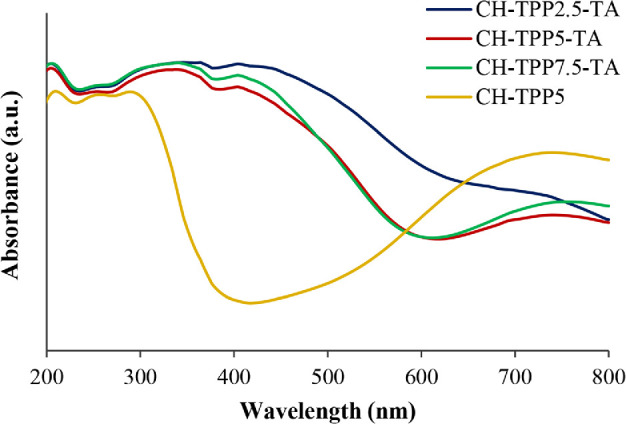
UV-Vis spectra of cryogels after Cu^2+^ ion adsorption.

The complexation mechanism of metal ions with ligands in cryogels was obtained by UV-Vis diffuse reflection spectroscopy. CH-TPP and CH-TPP-TA have quite similar spectra at wavelengths less than 300 nm (figure 5), due to the same band of amide bond and ligand-to-metal charge transfer (LMCT) of CH-Cu complexes. It tends to overlap some peaks of the TA compound. The main peak of the amide bond appears at a wavelength of 204−209 nm due to π–π* electron transitions of C=O. It slightly shifts from 214 nm, which is the peak of the CH reagent spectra in the various pH levels of 1−6 in solution (electronic supplementary material, figure S4a). In this area, absorption from the gallic acid chromophore, a TA component, is also possible. A gallic acid chromophore is usually found in the absorption band at approximately 210 nm [63]. The CH-Cu^2+^ complex was identified at a wavelength of 253−258 nm (figure 5). Another study reported that CH-Cu^2+^ peaks at approximately 243 nm [64].

TA-Cu^2+^ complex peak was found in all CH-TPP-TA cryogels at a wavelength of 337−346 nm (figure 5). It is slightly lower than the TA-Cu solution spectra at 375 nm, or there is a blue shift (electronic supplementary material, figures S4d and S5a). This peak is the LMCT band of TA-Cu^2+^, which generally occurs at approximately 344 nm [65]. In the TA-Cu solution, this peak begins to appear at pH 5 and 6 (electronic supplementary material, figure S4c) due to the ionization of the polyphenol structure of TA. TA-Cu^2+^ complexation involves the hydroxyl group of ellagic acid with Cu^2+^ [66]. Ellagic acid is one of the components of TA, together with gallic acid, flavone and phloroglucinol [67]. The shift and broadening of the cryogel adsorption band were detected clearly in the 740−754 nm region when compared with the CuCl_2_ solution spectra, which have a peak at 786 nm (electronic supplementary material, figure S5). In the CH-Cu^2+^ solution, this peak is formed at pH 3 or higher at approximately 790 nm (electronic supplementary material, figure S4b). This area is attributed to the d–d electronic transition [68]. The shift of the d–d transition to a lower wavelength at approximately 750 nm occurs because the ligand of CH enters the copper coordination sphere, which causes the 3d orbital of the copper ion to have varying energies [69,70].

The difference between the CH-TPP-TA and CH-TPP spectra after Cu^2+^ adsorption lies in the 404 nm peak (figure 5). It is the n–π* electron transition of the quinone compound [71]. During the adsorption process, it is likely that the hydrolysis and oxidation process of the TA compound occur to form a semi-quinone, then complete oxidation to form a quinone [72]. The quinone conjugation system causes the colour of the cryogel after adsorption to be dark yellow to dark brown. The spectra of CH-TPP2.5-TA have a slight difference in the wavelength region of 500−650 nm when compared with the spectra of CH-TPP5-TA and CH-TPP7.5-TA. We suspect that the absorption is derived from quinone compounds. It was previously found that some quinone derivatives have intense absorption at a wavelength of 500−600 nm [73]. The composition of TA in CH-TPP2.5-TA is higher than in CH-TPP5-TA and CH-TPP7.5-TA, which is 23.7% compared with 21.0% and 19.3% (electronic supplementary material, table S1). Therefore, the oxidation chance of TA to quinone in CH-TPP2.5-TA is greater than in other cryogels, leading to the spectral difference. In addition, the difference in Cu^2+^ ions concentration of 100, 200 and 500 mg l^−1^ in cryogel did not significantly affect the UV-Vis spectra (electronic supplementary material, figure S6a−d).

### 3.3. Effect of initial concentration of Cu^2+^ ions

**Figure 6.**
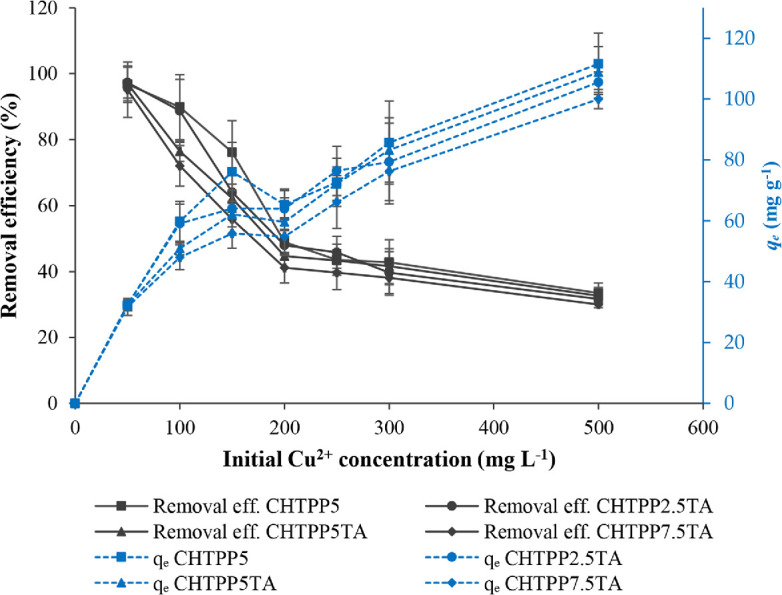
The effect of the initial concentration of Cu^2+^ ions on adsorption.

The influence of the initial Cu^2+^ ion concentration on *q*_e_ was evaluated (electronic supplementary material, table S4). The removal efficiency of Cu^2+^ ions decreased with increasing concentration (figure 6). Decreased available adsorption sites on the adsorbent cause this behaviour. At an initial Cu^2+^ ion concentration of 500 mg l^−1^, all cryogels’ formulae could still adsorb up to 30% Cu^2+^ ions. Cryogels remain capable of binding metal ions at high concentrations. They possess abundant active groups derived from CH or TA. This is evidenced by the *q*_e_ values that continue to increase even at high metal ion concentrations. This phenomenon can occur due to chemical and physical adsorption mechanisms [74].

The removal percentage of Cu^2+^ ions was inversely proportional to the *q*_e_ value. The adsorbed Cu^2+^ value increased with increasing initial Cu^2+^ concentration (figure 6). A high initial concentration of Cu^2+^ ions results in a high *q*_e_ value but a low percentage of ion removal. However, the cryogel does not reach saturation. All cryogels can adsorb Cu^2+^ ions at approximately 100−111 mg g^−1^, even though the initial concentration of Cu^2+^ ions has reached 500 mg l^−1^. Cryogel has a wide pore structure, so the adsorbate is easily transported through the pores. At that time, absorption and chemical reactions easily occur on the pore walls of the cryogel [36]. A higher concentration of Cu^2+^ ions increases the number of collisions with the cryogel and binds to more places in the cryogel; therefore, the *q*_e_ value increases [75]. This phenomenon is described in more detail in §3.7.

### 3.4. Effect of adsorption times

**Figure 7.**
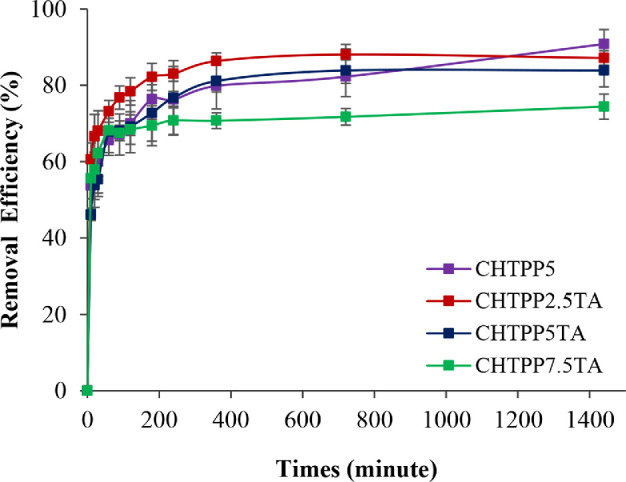
The effect of time adsorption on the adsorption efficiency.

The adsorption of Cu^2+^ ions with an initial concentration of 100 mg l^−1^ increased with increasing adsorption time. At 10 min, all cryogels had a Cu^2+^ ion removal efficiency of approximately 50% (figure 7; electronic supplementary material, table S5). The adsorption efficiency of Cu^2+^ by cryogels increased rapidly at the initial contact time and gradually equilibrated with increasing contact time. Rapid adsorption occurred within 30 min and reached the adsorption equilibrium after 360 min. At the beginning of the adsorption process, the cryogels have a large surface area to interact with metal ions. It also occurs due to the adsorption through the amine and hydroxyl groups of CH on the pores of the cryogel [49]. As the equilibrium time approaches, the adsorption sites are gradually filled by metal ions [76]. The performance of CH-TPP5, CH-TPP2.5-TA and CH-TPP5-TA cryogels to adsorb Cu^2+^ ions was quite similar in the 83–90% range. CH-TPP5 has a removal efficiency of up to 90% after 24 h of adsorption. However, based on previous research, cryogel without TA has a structure that quickly swells [30]. Again, we proved in this study that cryogel without TA experienced a more significant change in size than cryogel with TA. CH-TPP5 experienced a swelling of 38.9% from its initial size, while CH-TPP-TA was 8–13% (electronic supplementary material, figure S7). This proves that CH-TPP-TA can maintain its structure from swelling. Therefore, CH-TPP-TA is more effective in a continuous adsorption system than CH-TPP5. The polymer network of CH-TPP5 expands during swelling, resulting in the spread of the adsorbate. Furthermore, swelling causes the cryogel to soften, making it difficult to regenerate, leading to ineffective adsorption [77].

### 3.5. Adsorption isotherm

**Figure 8.**
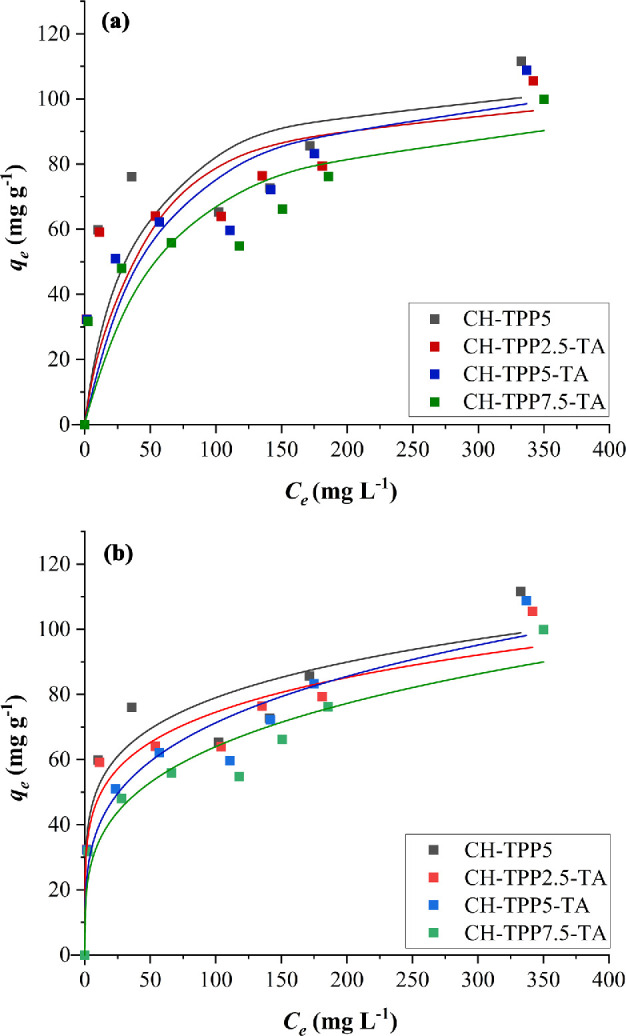
Langmuir (a) and nonlinear Freundlich (b) isotherm models of Cu^2+^ ion adsorption, filled squares (■) indicate *q*_e_ of the experiments and dashed lines (▬) indicate *q*_e_ of the isotherm models.

The adsorption isotherm was determined using five isotherm models, including Langmuir, nonlinear Langmuir, Freundlich, nonlinear Freundlich and Temkin models (figure 8; electronic supplementary material, figures S8–S11). The highest *R^2^* value indicates the selected isotherm model (table 2). Adsorption of Cu^2+^ ions in CH-TPP-TA was better described by the nonlinear Freundlich isotherm model, while CH-TPP5 by the Langmuir isotherm model (figure 8). Langmuir’s model identifies that metal ions are orderly adsorbed on the adsorbent’s homogeneous surface [1,78]. CH-TPP5 tends to have a homogeneous surface. Some modified CH are suitable for adsorbing metal ions fitting the Langmuir isotherm model [5]. The amine groups of CH are more dominant in binding metal ions. The complex formation between Cu^2+^ and the amine site of CH occurs in two stages. [Cu-(-NH_2_)]^2+^ begins to form and continues to the formation of [Cu-(-NH_2_)_2_]^2+^ [69]. This mechanism follows the bridge model, which describes the coordination bond between two amine groups with the Cu^2+^ ions [68,79]. The bridge model is a complex formation mechanism between a Cu^2+^ ion and CH in a tetra-coordination form. This model is predicted to have high coordination bond stability [80].

**Table 2.** Comparison of isotherm parameters.

isotherm models	parameters	CH-TPP5	CH-TPP2.5-TA	CH-TPP5-TA	CH-TPP7.5-TA
Langmuir	*q*_max_ (mg g^−1^)	109.4	105.4	112.1	103.8
	*K*_L_	0.033	0.031	0.022	0.029
	*R* ^2^	0.929	0.945	0.915	0.910
nonlinear Langmuir	*q*_max_ (mg g^−1^)	85.33	79.57	97.60	89.54
	*K*_L_	0.280	0.389	0.034	0.031
	*R* ^2^	0.852	0.846	0.792	0.786
Freundlich	*K*_F_	32.88	32.33	27.90	24.58
	1/*n*_F_	0.189	0.180	0.202	0.207
	*R* ^2^	0.847	0.905	0.9081	0.896
nonlinear Freundlich	*K*_F_	33.43	30.73	21.28	18.26
	1/*n*_F_	0.187	0.192	0.263	0.272
	*R* ^2^	0.913	0.947	0.945	0.943
Temkin	*B*	11.46	10.55	11.81	11.47
	*A*_T_	11.39	13.67	5.159	3.345
	*R* ^2^	0.784	0.827	0.769	0.759

CH-TPP-TA exhibits a different isotherm model influenced by the heterogeneous surface. The nonlinear Freundlich isotherm model describes that adsorbates are adsorbed on the heterogeneous surfaces of the adsorbent [81]. Heterogeneous surfaces of CH-TPP-TA came from many active sites, such as -NH_2_ and -OH of CH and -OH of TA. Their adsorption performances are different; therefore, they have many interactions with Cu^2+^ ions (adsorbate) [82]. The *k*_F_ value obtained in this study is relatively high (18–33 mg g^−1^). The *k*_F_ value explains the relationship between the Freundlich heterogeneous adsorbents and the *q*_e_ value [83]. A high *k*_F_ value means that the cryogel has good adsorption strength. The tailored CH/orange peel hydrogel has a *k*_F_ of 2.65 mg g^−1^ [84] and epichlorohydrin-triphosphate cross-linked CH-based adsorbs Cu(II) with a *k*_F_ of 20.76 mg g^−1^ [85]. We found that the values of 1/*n*_F_ of CH-TPP2.5-TA, CH-TPP5-TA and CH-TPP7.5-TA were 0.19, 0.26 and 0.27, respectively. They mean that the chemisorption mechanism dominates on the heterogeneous cryogel surface because chemisorption occurs when the 1/*n*_F_ value is less than unity [86,87]. The metal–ligand ion interactions on the cryogel surface involve active states of amine, hydroxyl and phosphate from CH, TA and TPP. Cu^2+^ ions and amines form a coordinate bond. The hydroxyl group of TA chelates Cu^2+^ ions. It probably forms a bidentate chelate bond where two oxygen atoms are bonded to the copper ion [88]. Meanwhile, the phosphate group of TPP also probably interacts ionically with the Cu^2+^ ion (electronic supplementary material, figure S1).

In this study, the *q*_e_ value of the cryogel using TPP up to 7.5% concentration (CH-TPP7.5-TA) did not differ significantly. However, there was a slight decreasing trend with the increase in TPP concentration. Using many TPP molecules in CH-TPP7.5-TA may inhibit the interaction of TA and CH. Therefore, TA of CH-TPP7.5-TA plays a lesser role in adsorbing metal ions than CH-TPP2.5-TA and CH-TPP5-TA. Due to the metal complexation strength and mechanical strength of CH, it must be considered to obtain superior materials. CH-TPP5-TA has a *q*_e_ value of 109 mg g^−1^ at an initial Cu^2+^ ion concentration of 500 mg l^−1^. This means the Cu^2+^ ions are adsorbed to approximately 1.72 mmol per gram of cryogel. Assuming that the binding structure of Cu^2+^ to amine of CH is the bridge model, the number of active sites of cryogel is calculated to be greater than or equal to 1.35 mmol g^−1^ (electronic supplementary material, table S1) because CH used in this study has a DD value greater than or equal to 80%, corresponding to greater than or equal to 4.3 mmol of amine group per gram of CH. Thus, more than 80% of the Cu^2+^ ions were first adsorbed by the amine active sites. Meanwhile, the rest of the adsorbed Cu^2+^ ions without binding to amine should be bound by the hydroxyl active sites of TA. TA will involve -OH groups from each phenolic group to chelate Cu^2+^. The number of phenolic groups in a TA is 10 units.

### 3.6. Adsorption kinetics

**Figure 9.**
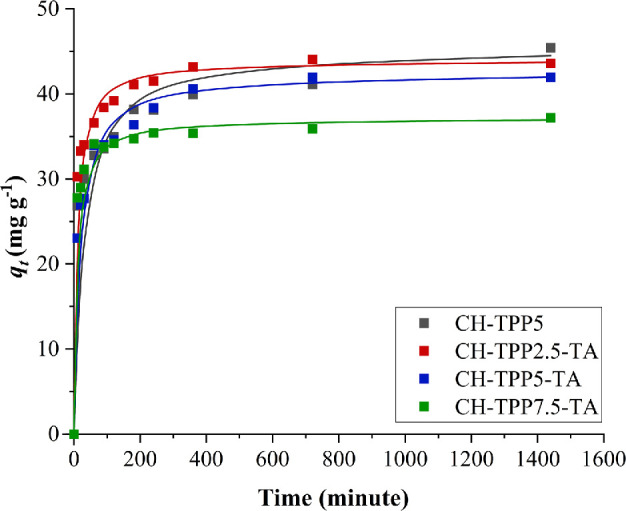
Pseudo-second-order kinetic of Cu^2+^ ion adsorption, filled squares (■) indicate *q_t_* of the experiments and dashed lines (▬) indicate *q_t_* of the PSO models.

The adsorption kinetic models tested were PFO and PSO (electronic supplementary material, table S6). Based on adsorption data at various adsorption times, it was found that the *R^2^* value of PSO was higher than PFO (electronic supplementary material, figures S12, S13). Thus, the kinetics of adsorption of Cu^2+^ ions by all cryogels follow the kinetics of PSO (figure 9). These results explain that Cu^2+^ ions are chemically bound to the cryogels.

PSO controls adsorption due to chemisorption mechanisms, such as covalent and complex bond formation [23]. PFO is more suitable for physisorption; these kinetics are more effective at high initial metal concentrations. Therefore, PFO occurs faster than PSO [5]. PSO also represents a strong coordination interaction between the metal ion and the adsorbent located in the cavity of the cryogels [1]. Several studies state that Cu^2+^ ion adsorption by CH more closely follows PSO kinetics. We found that the *q*_e_ value on PSO kinetics for CH-TPP, CH-TPP2.5-TA and CH-TPP5-TA cryogels was more than 40 mg g^−1^, while CH-TPP7.5-TA was less than 40 mg g^−1^ (table 3). Using TPP with a high concentration reduces the *q*_e_ value. We estimated that from the use of 2.5, 5, to 7.5% of TPP, the amounts of active sites of TA in the cryogel continued to decrease, namely 1.39, 1.23 and 1.13 mmol g^−1^ cryogel, respectively (electronic supplementary material, table S1). The high TPP composition also reduces the levels of CH amine sites. The calculation results show that the levels of amine sites of CH-TPP2.5-TA, CH-TPP5-TA and CH-TPP7.5-TA were 1.45, 1.35 and 1.25 mmol g^−1^ cryogel, respectively (electronic supplementary material, table S1). Therefore, the performance of cryogels to Cu^2+^ ion complexes also decreases.

**Table 3.** Comparison of pseudo-first order and pseudo-second order kinetics.

cryogels	pseudo-first order			pseudo-second order		
	*q*_t_ (mg g^−1^)	*k*_1_ (min^−1^)	*R* ^2^	*q*_t_ (mg g^−1^)	*k*_2_ (g mg^−1^ min^−1^)	*R* ^2^
CH-TPP5	17.320	0.0025	0.6527	45.419	0.0007	0.9975
CH-TPP2.5-TA	15.984	0.0085	0.8665	44.004	0.0023	0.9999
CH-TPP5-TA	19.965	0.0076	0.8904	42.534	0.0013	0.9997
CH-TPP7.5-TA	6.9714	0.0031	0.4857	37.197	0.0027	0.9997

### 3.7. Cryogel morphology and photograph after adsorption

**Figure 10.**
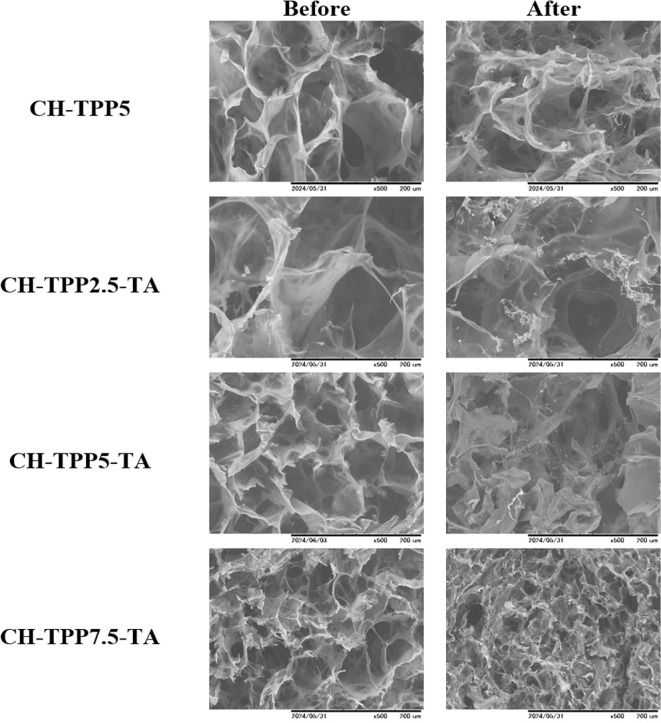
Morphology of cryogels before and after adsorption of Cu^2+^ ions.

The comparison of cryogel before and after adsorption was investigated using its morphology. All cryogels used for Cu^2+^ ion adsorption have apparent differences in the cryogel structure (electronic supplementary material, figure S14). CH-TPP2.5-TA and CH-TPP5-TA have a supermacropore structure that looks different from the CH-TPP7.5-TA cryogel. The morphology of the cryogel after adsorption was obtained after drying (figure 10). The dry cryogel structure after metal ion adsorption appeared to experience changes in the pore structure, especially in the CH-TPP7.5-TA cryogel. This pore damage occurred because the cryogel structure was stiffer when using high TPP concentrations than at low concentrations. As explained in the mechanical properties of the cryogel, decreased cryogel elasticity will impact the fragility of the cryogel’s pore structure.

**Figure 11.**
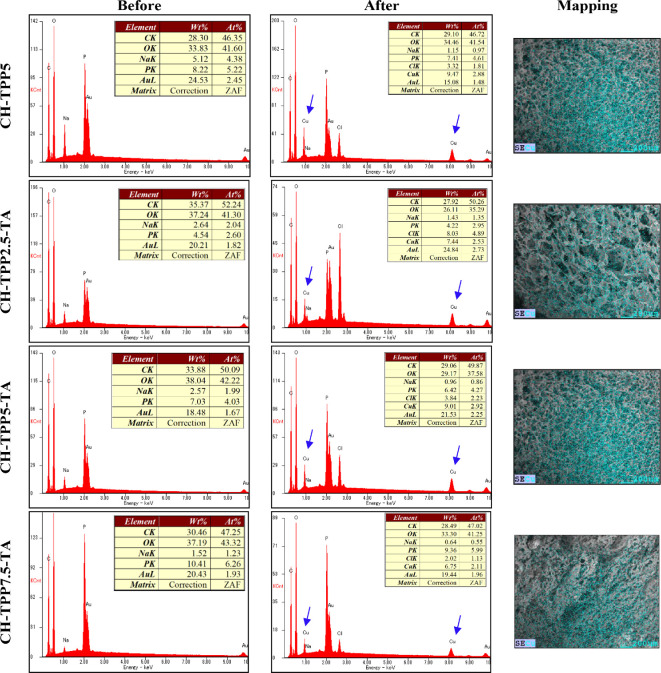
EDX spectrum before and after adsorption of Cu^2+^ ions and mapping of Cu^2+^ ions on cryogels.

The Cu^2+^ ions adsorbed in the cryogels were investigated using EDX. Analysis using SEM-EDX begins with coating the cryogels with gold. The purpose of this coating is to prevent the sample from charging up during observation scanning. EDX data clearly show the differences in cryogels before and after adsorption. Before adsorption, Cu was not detected, whereas after adsorption, Cu was found in the cryogels (figure 11). The distribution of Cu^2+^ ions on the cryogel surface is shown from EDX data mapping. These results have proven the adsorption of Cu^2+^ ions by the cryogel. The chlorine (Cl) element comes from CuCl_2_ and is an adsorbate solution.

**Figure 12.**
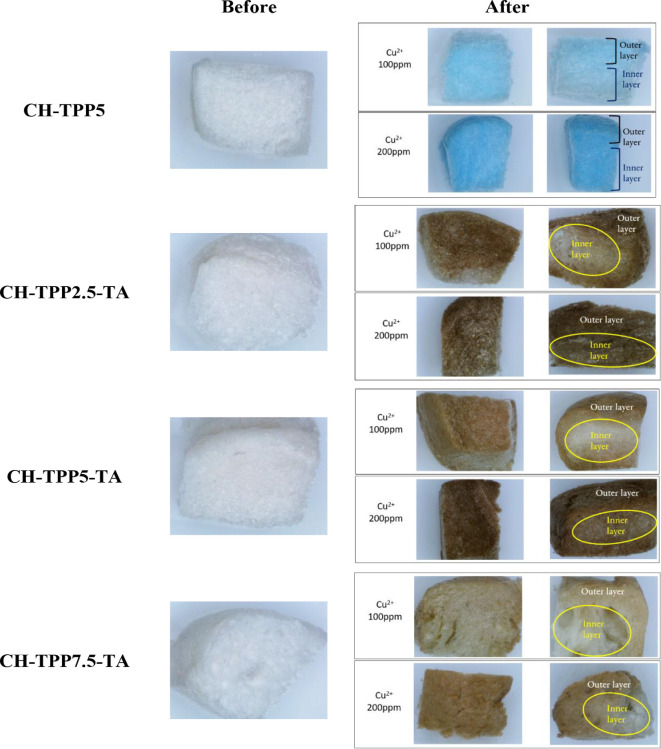
Photograph of cryogels surface before and after adsorption of Cu^2+^ ions (magnification indication of 0.8).

**Figure 13.**
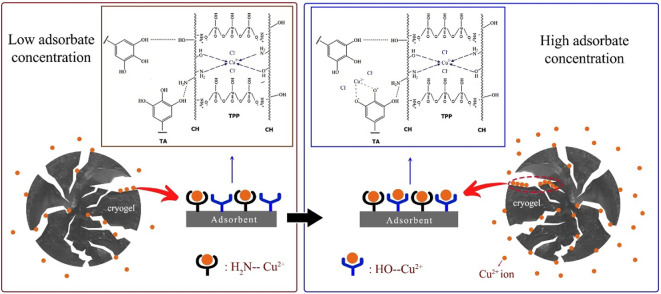
Mechanism of Cu^2+^ ion adsorption at different concentrations in the CH-TPP-TA cryogel.

The adsorption results were accompanied by photographs using a stereomicroscope system (figure 12). The appearance of the four cryogels looks different. CH-TPP5 produces a bright blue colour due to the complexation of Cu^2+^ ions and CH. In CH-TPP-TA, the colour of the cryogels is brown due to the complexation of Cu^2+^ ions with CH and TA (figure 12). The brown colour of the cryogel fades with increasing TPP concentration in the cryogel because CH interacts more with TPP than with TA; therefore, the TA-Cu complex is reduced. The UV-Vis spectrum in figure 5 explains the mechanism of this complexation. CH-TPP involves amine groups on the CH backbone to complex Cu^2+^ ions, producing a light blue material. In contrast, CH-TPP-TA is assumed to involve hydroxyl groups of TA and amine groups of CH to complex Cu^2+^ ions, indicated by the cryogel’s dark brown colour. Photography was also carried out at two concentrations of Cu^2+^ ions of 100 and 200 ppm. Generally, the adsorption of Cu^2+^ 100 ppm by the four cryogels was more dominant around the outer layer than inside the cryogels. However, at Cu^2+^ 200 ppm adsorption process, it can occur up to the inner layer of the cryogels (figure 12). The heterogeneous cryogel surface also plays an essential role in the adsorption of metal ions with different concentrations. The amine group of CH will be more active in binding metal ions at low concentrations. In contrast, when the metal ion concentration is high, the hydroxyl group of TA will actively complex metal ions (figure 13). The abundance of active sites on CH-TPP-TA has supported the absorption of metal ions to the deepest layer of the cryogel.

### 3.8. Comparison with the other study

The adsorption capacity obtained in this study was generally higher than other adsorbents based on CH or TA (table 4). The CH-TPP-TA cryogel had higher adsorption capacity (109 mg g^−1^) compared with glutaraldehyde cross-linked CH cryogel (61.1 mg g^−1^), CH-cellulose hydrogel (94.3 mg g^−1^), magnetic Fe_2_O_3_-CH (53.3 mg g^−1^) and TA-hydroxyapatite (94.8 mg g^−1^). The abundant amine and hydroxyl groups of CH-TPP-TA cryogel play an essential role in the adsorption of Cu^2+^ ions. The supermacropore structure of the cryogels increases the ability of the CH-TPP-TA composite to adsorb metal cations. Furthermore, its biocompatible property provides advantages for use in water treatment. It can be used directly in drinking water treatment without worrying about the toxicity effects on humans. This cryogel is believed to be a superior material for the future.

**Table 4.** The adsorption capacity of Cu^2+^ ions on CH-TPP-TA cryogels compared with other adsorbents.

adsorbent	*q*_m_ (mg g^−1^)	references
chitosan-clinoptilolite-glutaraldehyde cryogel	61.1	[89]
mesoporous cellulose-chitosan hydrogel	94.3	[90]
magnetic microspheres of chitosan-SA-PVA-Fe_3_O_4_	58.3	[46]
chitosan-glutaraldehyde-methyl methacrylate	192.3	[91]
chitosan modified with carboxyl groups	220.5	[92]
chitosan-conjugated iodate porous	95.2	[93]
tannic acid–nano-hydroxyapatite	94.8	[94]
chitosan-TPP-TA cryogel	109^a^	this study

^a^ It is a *q*_e_ value from the experiment data at Cu^2+^ ion initial concentration of 500 mg l^−1^.

## 4. Conclusion

This study successfully synthesized a biocompatible adsorbent using cryogel from CH, TPP and TA. TPP and TA act as biocompatible cross-link agents. A high concentration of TPP (7.5 wt%) has a brittle dry cryogel structure and a lower Cu^2+^ ion adsorption effectiveness than TPP 2.5 and 5%. The *q*_e_ value was obtained from the CH-TPP5-TA cryogel of 109 mg g^−1^ at a temperature of 303 K, pH 5.0, an initial Cu^2+^ ion concentration of 500 mg l^−1^ and an adsorption time of 24 h. Adsorption of Cu^2+^ ions in the CH-TPP-TA cryogel fits the nonlinear Freundlich isotherm model and pseudo-second-order kinetics. These results identify that Cu^2+^ ions are mainly adsorbed on the heterogeneous surfaces of the cryogel. They interact chemically (chemisorption) with the cryogel through coordination bond, ionic interaction and complex formation. Furthermore, Cu^2+^ ions have been adsorbed into the inner layer of the cryogel. Using TA in CH cryogel has the same ability as cryogel without TA for Cu^2+^ ions metal adsorption. However, TA has the advantage of increasing the mechanical properties of cryogel and resistance to acid solutions. The good mechanical properties of the cryogel make it suitable for recycling the cryogel and application in continuous adsorption systems. This system is superior for large-scale adsorption due to its low process cost. Overall, CH-TPP-TA cryogel is promising to be used as a biocompatible adsorbent for copper ions. It is safe to be used directly for drinking water treatment.

## Data Availability

The data supporting this article have been included in the electronic supplementary material [95].
